# Viewing SARS-CoV-2 Nucleocapsid Protein in Terms of Molecular Flexibility

**DOI:** 10.3390/biology10060454

**Published:** 2021-05-21

**Authors:** Tatsuhito Matsuo

**Affiliations:** 1 Institute for Quantum Life Science, National Institutes for Quantum and Radiological Science and Technology, 2-4 Shirakata, Tokai-mura, Naka-gun, Ibaraki 319-1106, Japan; matsuo.tatsuhito@qst.go.jp; 2 Laboratoire Interdisciplinaire de Physique (LiPhy), Grenoble-Alpes University, 140 Rue de la Physique, 38402 Saint Martin d’Hères, France; 3 Institut Laue-Langevin, 71 Avenue des Martyrs, CS 20156, CEDEX 9, 38042 Grenoble, France

**Keywords:** coronavirus nucleocapsid protein, molecular flexibility, protein structure, protein dynamics, liquid-liquid phase separation, neutron scattering

## Abstract

**Simple Summary:**

Nucleocapsid protein is one of the essential proteins for viral replication including the coronavirus SARS-CoV-2, which causes coronavirus disease 2019 (COVID-19) pneumonia leading to the ongoing pandemic. Whereas this protein has emerged as a potential drug target, its physicochemical properties and the molecular mechanism of how this protein is involved in viral replication remain unclear. In this review, structural and dynamical aspects of the SARS-CoV-2 nucleocapsid protein (N^CoV2^), which should play a key role in a new drug development and in the interaction with RNA and other proteins are summarized. The structural feature of N^CoV2^ is first described. Next, simulation studies on the interaction between potential drug molecules and N^CoV2^ are summarized, which show the importance of molecular flexibility. Then, liquid-liquid phase separation phenomenon involving N^CoV2^, which has recently been reported, is described. This phenomenon also suggests the importance of molecular flexibility of N^CoV2^. Finally, a promising method using neutron scattering to characterize the structure and structural fluctuation of the droplets, which are formed through this phenomenon, is presented.

**Abstract:**

The latest coronavirus SARS-CoV-2, which causes coronavirus disease 2019 (COVID-19) pneumonia leading to the pandemic, contains 29 proteins. Among them, nucleocapsid protein (N^CoV2^) is one of the abundant proteins and shows multiple functions including packaging the RNA genome during the infection cycle. It has also emerged as a potential drug target. In this review, the current status of the research of N^CoV2^ is described in terms of molecular structure and dynamics. N^CoV2^ consists of two domains, i.e., the N-terminal domain (NTD) and the C-terminal domain (CTD) with a disordered region between them. Recent simulation studies have identified several potential drugs that can bind to NTD or CTD with high affinity. Moreover, it was shown that the degree of flexibility in the disordered region has a large effect on drug binding rate, suggesting the importance of molecular flexibility for the N^CoV2^ function. Molecular flexibility has also been shown to be integral to the formation of droplets, where N^CoV2^, RNA and/or other viral proteins gather through liquid-liquid phase separation and considered important for viral replication. Finally, as one of the future research directions, a strategy for obtaining the structural and dynamical information on the proteins contained in droplets is presented.

## 1. SARS-CoV-2 Nucleocapsid Protein

A new coronavirus named severe acute respiratory syndrome coronavirus 2 (SARS-CoV-2) has caused the cataclysmic pandemic through coronavirus disease 2019 (COVID-2019) pneumonia. SARS-CoV-2 is a member of β-coronavirus family that includes well-known SARS-CoV and MERS-CoV [1]. It is an enveloped, single-stranded, positive-sense RNA virus whose genome of 30 kb is packaged into a virion of ~90 nm in diameter, which is a similar size as that of other coronaviruses [2]. SARS-CoV-2 consists of 29 proteins in total: 16 non-structural proteins (nsp1–nsp16), 9 accessory proteins (Orf3a, Orf3b, Orf6, Orf7a, Orf7b, Orf8, Orf9b, Orf9c and Orf10) [3], and 4 major structural proteins, which are the spike (S) protein, the envelope (E) protein, the membrane (M) protein and the nucleocapsid (N) protein. SARS-CoV-2 infection is initiated through binding of the spike protein to the angiotensin-converting enzyme 2 (ACE2) receptor in lung cells [4]. After infection, the envelope protein serves as a cation-selective channel across the endoplasmic reticulum–Golgi intermediate compartment (ERGIC) membrane [5]. The membrane protein is important for determination of the shape of the virus envelope [6]. It interacts with all the above structural proteins and promotes viral assembly by stabilizing the nucleocapsid-RNA complex (ribonucleocapsid or ribonucleoprotein; RNP). The membrane and envelope proteins are thus essential for virion assembly and budding. Among the proteins expressed in this virus, the nucleocapsid protein is one of the abundant viral proteins, which shows multiple functions including packaging the viral genome into a helical RNP. It is essential for transcription of the viral genome and virion replication. Therefore, much attention has been paid to the molecular mechanism of biological functions of N protein during the infection cycle. There have already been brilliant reviews on the coronavirus nucleocapsid proteins [7,8,9]. Even though the biochemical processes inside the infected cells that nucleocapsid protein is involved in and the high-resolution structural analysis by crystallography and cryo-EM have been featured, its structural properties and dynamical behavior in aqueous solution have not been addressed so far albeit its importance. In the following, the focus is put on both the structure and molecular flexibility of SARS-CoV-2 nucleocapsid proteins (N^CoV2^).

**Figure 1:**
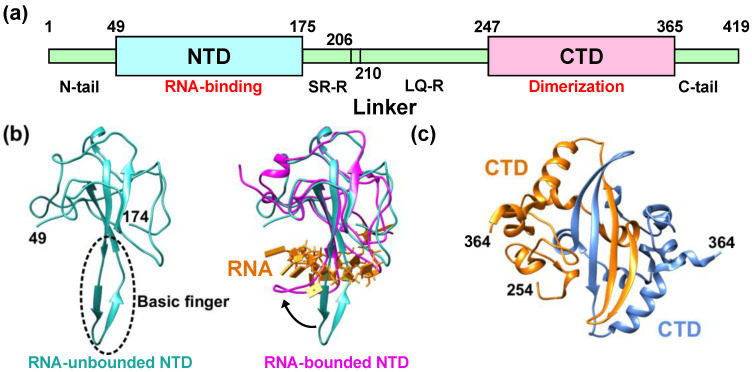
Structural features of the nucleocapsid protein of SARS-CoV-2 (N^CoV2^). (**a**) Schematic of N^CoV2^, which consists of two domains, i.e., the N-terminal domain (NTD) and the C-terminal domain (CTD); (**b**) atomic structure of the NTD in the RNA-unbounded form (left; PDB ID: 6M3M [12]) and the single-stranded RNA-bounded form (right; PDB ID: 7ACT [13]) are shown in light sea green and magenta, respectively. In the right panel of (**b**), RNA is denoted in orange and the RNA-unbounded form of the NTD is superimposed for structural comparison. The direction of the movement of the basic finger upon RNA-binding is shown by a black arrow; (**c**) CTD dimer interface. The CTD monomers (PDB ID: 6WZO [15]) are shown in orange and marine blue, respectively. In (**b**,**c**), the residue numbers at both termini of the structures that are visible in the crystal structures are denoted. All the models are depicted using UCSF Chimera [16].

Schematic illustration of N^CoV2^, which is composed of 419 residues in total, is shown in Figure 1a. N^CoV2^ consists of two domains, the N-terminal domain (NTD; 49–174) and the C-terminal domain (CTD; 247–364). The NTD is responsible for RNA-binding and the CTD for dimerization and oligomerization (note that in the case of the nucleocapsid protein of SARS-CoV, both domains have the ability to bind RNA molecules [10]). These domains are connected by a flexible linker, which contains Serine/Arginine-rich region (SR-R; 176–206) and Leucin/Glutamine-rich region (LQ-R; 210–246). Serine and arginine residues account for 45.2% and 19.4% of the total number of residues of the former linker, respectively, which is phosphorylatable in multiple sites. In LQ-R, leucine and glutamine account for 14.9% and 12.8%, respectively. In another nucleocapsid protein VP39 of baculovirus, it is known that amino acid substitution at glycine 276 affects nucleocapsid assembly and that this residue is completely conserved among various baculovirus families [11], suggesting that certain glycine residues are essential for proper function of the nucleocapsid protein. Even though N^CoV2^ contains 43 glycines in total, which account for 10.3% of 419 residues, there have not been systematic studies on the role of glycine and thus the possible roles of glycine in the N^CoV2^ function remain unclear. In addition to SR-R and LQ-R, the NTD and CTD are flanked by two disordered regions, i.e., the N-tail (1–48) and the C-tail (365–419), respectively. The atomic structures of the NTD have recently been solved by X-ray crystallography at 2.7 Å resolution [12] (Figure 1b left) and by nuclear magnetic resonance (NMR) [13]. Its feature is “(loops)-(β-sheet core)-(loops)” fold, which is conserved among the coronavirus NTDs. Similar to another coronavirus such as infectious bronchitis virus (IBV) [14], the protruding positively-charged β-hairpin structure called “basic finger” (the bottom of the left panel of Figure 1b) is essential for RNA-binding [13]. Upon single-stranded RNA-binding, the basic finger changes its conformation such that the NTD grabs the RNA molecule (Figure 1b right).

The crystal structures of the CTD have been solved at 1.4 Å [15,17] and 2.0 Å [18] resolutions. It has been shown that the dimer interface consists of two β-strands and one α-helix (Figure 1c), which is similar to that of the nucleocapsid protein of SARS-CoV [19]. In solution, the N^CoV2^ molecules have been shown to exist in dimers by small-angle X-ray scattering [20]. Note that, however, N^CoV2^ can also form tetramers [15], suggesting that its solution structure is sensitive to the solvent conditions. Whereas the N^CoV2^ dimers are formed through binding of two CTDs, the architecture of the tetramers remains unclear.

**Figure 2:**
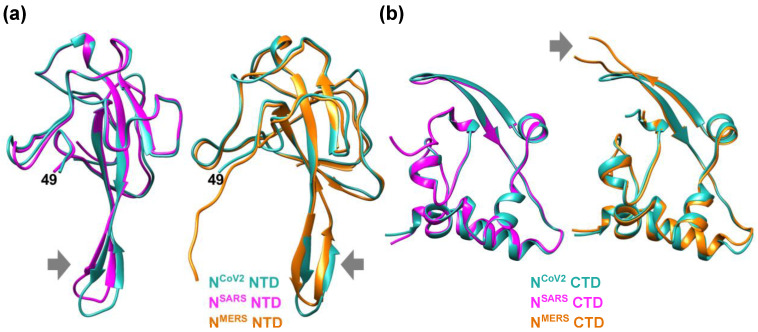
Comparison of the NTD and CTD structures of the nucleocapsid protein of different coronaviruses. (**a**) (left) Comparison of the NTD structures of the nucleocapsid protein of SARS-CoV-2 (N^CoV2^; PDB ID: 6M3M [12]) and SARS-CoV (N^SARS^; PDB ID: 2OFZ [21]), which are shown in marine blue and magenta, respectively. (right) Comparison of N^CoV2^ NTD and the NTD of the nucleocapsid protein of MERS-CoV (N^MERS^; PDB ID: 4UD1 [22]) shown in orange. Note that N^CoV2^ NTD in the left and right panels are displayed at slightly different angles to focus on the structural differences from the N^SARS^ and N^MERS^ counterparts; (**b**) (left) Comparison of the CTD structures of N^CoV2^ (PDB ID: 7C22 [18]) and of N^SARS^ (PDB ID: 2CJR [23]), which are shown in marine blue and magenta, respectively. (right) Comparison of N^CoV2^ CTD and the CTD structure of N^MERS^ (PDB ID: 6G13 [24]) shown in orange. In both (**a**,**b**), the major structural differences are shown by grey arrows. All the models are depicted using UCSF Chimera [16].

In order to see similarities and differences among different nucleocapsid proteins of coronavirus, the NTD and CTD structures of N^CoV2^ were compared with those of the nucleocapsid protein of representative coronaviruses SARS-CoV (N^SARS^) and MERS-CoV (N^MERS^) as shown in Figure 2. Even though the overall architecture is similar among them, superposition of the NTDs of N^CoV2^ and N^SARS^ (Figure 2a) shows that the β-hairpin structure of N^SARS^ NTD moves toward the N-terminus compared with N^CoV2^ NTD. On the other hand, when compared with N^MERS^ NTD (Figure 2b), the β-hairpin structure is more extended for N^MERS^ NTD. These comparisons suggest that the major differences in the NTD structure among the three coronaviruses reside in the β-hairpin structure, which is necessary for RNA-binding. These differences also imply that each coronavirus might have adapted the NTD structure to match its specific RNA conformation. Regarding the CTD structure, it is found that the structural feature is similar between N^CoV2^ CTD and N^SARS^ CTD. However, in the case of N^MERS^ CTD, the loop between the two β-sheets (indicated by a grey arrow in Figure 2b) is longer than the other CTDs. According to hydrogen/deuterium exchange measurements on dimers of N^CoV2^ CTD [15], this region is exposed to the solvent, suggesting that this region is not involved in dimer formation. So far, it is unclear what role this long insertion observed in N^MERS^ CTD plays in the function of the protein. The fact that other regions of the CTD possess common structural features among three coronaviruses displayed here suggests that dimer formation of the nucleocapsid protein through its CTD is essential for formation of RNP complexes and hence RNA genome packaging, which is the fundamental function of the nucleocapsid protein.

## 2. Molecular Flexibility and Drug Binding of N^CoV2^

The nucleocapsid protein, together with the spike and membrane proteins, has been used in vaccine development due to its high immunogenicity [25,26]. Furthermore, since the N^CoV2^ gene is more conserved, more stable and less mutated than other viral proteins such as the spike protein, it has also attracted attention as a potential drug target [27]. It is now well accepted that in the ligand/drug binding process, intramolecular flexibility of proteins such as the one in the active site and its surrounding regions plays a key role: these regions undergo conformational changes so that the structure in these sites matches the shape of the ligand/drug molecule, which is known as the “induced-fit” model [28]. Another mechanism is the “conformational selection” model [29,30], where a conformational change of proteins occurs prior to the binding of a ligand/drug molecule, meaning that the binding of other molecules shifts the equilibrium between the ground state (apo-form) and the excited state (holo-form). In any case, there exist structural fluctuations within protein molecules driven by thermal energy and the above mechanisms emphasize the importance of the structural dynamics of protein molecules such as thermal motions of amino acid side chains and backbones to understand the molecular basis of the ligand/drug binding to protein molecules and the stability of protein-ligand complexes. Therefore, it is essential to investigate the binding between a potential drug molecule and the RNA-binding site of N^CoV2^ taking account of the intramolecular flexibility. In this respect, molecular dynamics (MD) simulation technique demonstrates its power.

**Figure 3:**
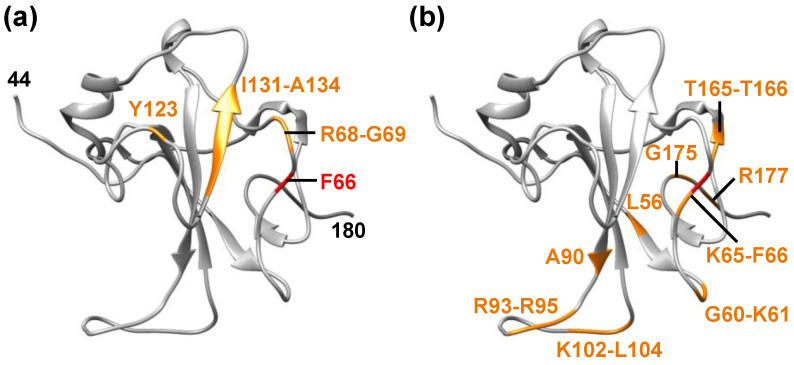
Drug binding sites and RNA-binding sites on N^CoV2^ NTD. The RNA-bounded form of the NTD is shown (PDB ID: 7ACT [13]). (**a**) Amino acid residues of N^CoV2^ NTD involved in the interaction with antiviral compounds that show high binding affinity are shown in orange (F66, R68, G69, Y123, I131, W132, V133 and A134 [31]). These residues commonly interact with the compounds. The residue numbers at both termini that are visible in the structure are denoted in black; (**b**) amino acid residues of N^CoV2^ NTD involved in RNA-binding are shown in orange (L56, G60, K61, K65, F66, A90, R93, I94, R95, K102, D103, L104, T165, T166, G175 and R177 [13]). F66 is the common residue between (**a**,**b**), and is shown in red. These models are depicted using UCSF Chimera [16].

In a recent study, combination of molecular docking and MD simulation was employed to identify potential antiviral drugs and investigate the stability of the NTD-drug complexes [31]. 34 antiviral compounds, which were already approved or in development, were focused on because such molecules do not need long-term preclinical studies and thus, they would be excellent candidates in the case of disease outbreaks. Since the location of the ligand binding site in the NTD of N^CoV2^ was not known, blind docking approach was used to predict the possible ligand binding sites. As a result, it was found that rapamycin has the best binding affinity to the NTD (binding energy: −11.9 kcal/mol). Other compounds called saracatinib, camostat, trametinib and nafamostat were also found to show high binding affinity (binding energy between −10.4 kcal/mol and −9.3 kcal/mol). These compounds are antiviral drugs used for the treatment of diseases caused by MERS-CoV (all the five compounds), SARS-CoV (trametinib) and another human coronavirus HCoV-229E (camostat). In addition, it was shown that the interactions of these compounds with the ligand-binding site on the NTD were slightly different from each other. The common amino acid residues of the NTD involved in the binding with these compounds are shown in Figure 3a, together with amino acid residues essential for RNA-binding (Figure 3b). This comparison shows that the drug-binding sites and RNA-binding sites share only one residue (F66) and that the above compounds mainly bind to the different sites from those for RNA-binding. Ivermectin, an anti-parasitic drug with antiviral properties, has also been shown to bind to the similar region as the above compounds [32]. Although each compound also interacts with the residues of the NTD other than those shown in Figure 3a, none of these residues overlaps with the RNA-binding region except for rapamycin, which shares K65 with the RNA-binding sites [31]. It is likely that the binding affinity between the NTD and RNA is affected as long as at least one residue is blocked by a drug molecule.

After identifying the above compounds that bind to the NTD with high affinity, the stability of the NTD-drug complexes was investigated using MD simulation at 300 K for 25 ns [31]. It was found that the root-mean-square-fluctuation (RMSF), which reflects the magnitude of thermal motions of amino acid residues, of all the complexes is between 0.42 nm and 1.32 nm, suggesting that the complexes are highly stable. This also suggests that the interactions between amino acid side chains that play an important role in the binding affinity between the NTD and all the compounds remain stable. Intriguingly, the RMSF values of the basic finger of the NTD were enhanced in the complexes with camostat, trametinib and nafamostat compared with the complexes with rapamycin and saracatinib despite the fact that none of these compounds directly interacts with the basic finger. This suggests that drug-binding-induced changes in the structural fluctuation of the residues in the drug binding sites are transmitted to and amplified at a distant region. Since the basic finger is essential for RNA-binding [13], it is possible that the enhanced fluctuation in the basic finger destabilizes the interaction between the NTD and RNA, which points to the importance of molecular flexibility in modulating the NTD-RNA interaction.

Even though most studies based on MD simulation have targeted the NTD, there is a unique study targeting the CTD to identify potential antiviral drugs to prevent dimer or oligomer formation [33]. In this study, the binding affinity and stability of five compounds 4E1RCat, silmitasertib, TMCB, sapanisertib and rapamycin with N^CoV2^ CTD were investigated. It was found that 4E1RCat shows the highest affinity (−10.95 kcal/mol) while other compounds also show relatively high affinity between −8.91 kcal/mol and −6.14 kcal/mol. These compounds bound to the dimeric interface region of the CTD, suggesting that the drug molecules directly hamper dimerization of the CTDs. Furthermore, the results of the MD simulation on the CTD-drug complexes showed that drug binding affects the secondary structure of the CTD: the drugs silmitasertib and rapamycin increased the α-helical and β-sheet contents while the other drugs (4E1RCat, TMCB and sapanisertib) induced the loss of β-sheet contents and thus decreased the rigidity of the protein-drug complex. However, whereas the overall structural fluctuation measured by RMSF was decreased upon drug binding for all the CTD-drug complexes, the magnitude of the decrease in RMSF was smaller for 4E1RCat and TMCB than for other drugs. The expected increase in RMSF due to partial collapse of the secondary structures upon the drug binding was not observed, perhaps due to the rigidification of part of the protein molecules caused by binding of 4E1RCat and TMCB, and the overall effect of the drug binding was a decrease in RMSF. Knowledge of the dynamical aspect of proteins thus provides important information to understand the detailed mechanism of the interaction between protein and ligand/drug.

Another study, which employed a similar approach as above, focused on compounds from medicinal plants [34]. Five compounds, aloe-emodin, anthrarufin, alizarine, dantron and emodin, were successfully singled out from 100 phytocompounds that show high binding affinity with the NTD. Thus, various compounds from different sources have extensively been tested to discover new therapeutic agents for SARS-CoV-2 using MD simulation. Together with other relevant studies [35,36,37], MD simulation, which incorporates structural fluctuations into simulations, plays a crucial role in drug screening and is paving the way for a new drug development.

In addition to the intramolecular flexibility described above, there is another important property that affects the binding process between a drug/RNA and N^CoV2^, which is the interdomain flexibility of N^CoV2^. The NTD is connected to the CTD by a flexible linker region, which in general is assumed to be in the disordered state. According to the research during the last decades, however, intrinsically disordered regions are not completely in random coils [38,39,40,41], but are in equilibrium between the random coils and partially folded structures taking on α-helices, the relative population of which varies between 10% and 70% depending on proteins [42]. In the case of N^CoV2^, while 42% of the residues of the central linker (175–246) takes on transient α-helices, the frequency of which depends on whether the linker is in isolation or connected to the NTD and CTD [43], suggesting that the helical propensity in the flexible region is affected by the local environment. Physiological substances such as osmolytes are also known to affect the structural properties of flexible regions [44], pointing to the importance of the physicochemical environment in which the proteins are located. The chemical composition inside the cells is known to change according to the cell-cycle stage and by external stress [45,46,47,48,49,50]. This raises the possibility that the interdomain linker of N^CoV2^ takes various forms with different flexibility in the infected cells depending on the chemical environments, which would affect the mobility of each domain. Therefore, in terms of a new drug development, it would be informative to know what effects the interdomain flexibility could have on the drug encounter/binding rate. However, there was no study focusing on this aspect.

**Figure 4:**
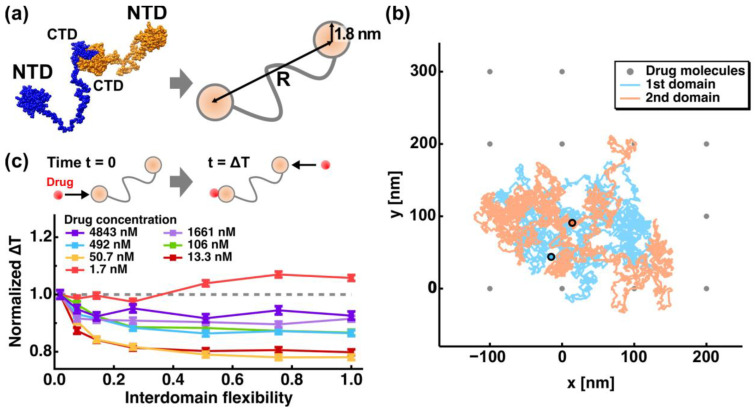
Dependence of the interdomain flexibility of N^CoV2^ on the drug encounter rate. (**a**) Approximation of the atomic structure of N^CoV2^ by two circles with corresponding radii. The interdomain flexibility is represented by the range of distance (R) between the two NTDs; (**b**) example of a trajectory of random walk simulation of the N^CoV2^ model. The initial position of the two NTDs are shown in cyan and orange. The grey points denote the positions of the drug molecules located every 100 nm corresponding to the drug concentration of 1.7 nM; (**c**) the time interval (ΔT) between the first drug binding to one NTD, which occurs at time t = 0, and the second drug binding to the other NTD at various drug concentrations and at various values of interdomain flexibility. In this graph, the lowest flexibility defined as 19.0 (nm) ≤ R ≤ 21.0 (nm) and the highest flexibility defined as 3.6 (nm) ≤ R ≤ 109.6 (nm) correspond to 0.02 and 1.0 on the abscissa labeled as “Interdomain flexibility”, respectively. All these values are normalized by the ΔT values obtained at the lowest flexibility, which were 72 ns, 257 ns, 678 ns, 2297 ns, 4358 ns, 11,856 ns and 27,062 ns at 4843 nM, 1661 nM, 492 nM, 106 nM, 50.7 nM, 13.3 nM and 1.7 nM, respectively. These figures are taken and adapted from [51] with permission.

Recently, effects of the interdomain flexibility on the drug encounter/binding rate have been investigated using two-dimensional random walk simulation [51]. In this study, N^CoV2^ homodimer was approximated by a structure where two NTDs, each of which possesses the RNA-binding site and thus binds a drug molecule, are connected by a long flexible linker as schematically shown in Figure 4a. Hereafter, this linker connecting the two NTDs is simply called an interdomain linker. The NTDs were approximated by two circles with a corresponding radius of 1.8 nm. The interdomain flexibility was represented as the range of the distance (R as shown in Figure 4a) between the two NTDs, i.e., R_min_ (nm) ≤ R (nm) ≤ R_max_ (nm), where R_min_ and R_max_ are the minimum and maximum R values, respectively. The lowest flexibility was defined as 19.0 (nm) ≤ R ≤ 21.0 (nm) while the highest flexibility was defined as 3.6 (nm) ≤ R ≤ 109.6 (nm). Drug molecules were represented as lattice points as shown in Figure 4b, the interval of which was determined by the given drug concentrations. Drug concentrations were within the in vivo range between 1 nM and 5000 nM [52]. Validity on the assumption that drug molecules are located at fixed positions is discussed in the original paper [51]. The encounter between an NTD and a drug molecule, where the distance between them is equal to or less than 1.8 nm, was considered as “binding” because the affinity between the NTDs and drug molecules was not taken into account in the simulation. An example of a trajectory of the random walk simulation of the N^CoV2^ model is shown in Figure 2b. By changing R_min_, R_max_ (interdomain flexibility), and drug concentrations, the time interval between the first drug binding to one NTD and the second drug binding to the other NTD (ΔT) was calculated from 6000 trajectories. For each trajectory, 5000 steps of random-walk were conducted with each step corresponding to 21 ns (102 μs in total). The ΔT values normalized by those at the lowest interdomain flexibility are shown in Figure 4c as a function of interdomain flexibility at various drug concentrations. The decrease in ΔT at higher interdomain flexibility (*p* < 0.001 according to the Steel-Dwass test [53,54]) shows that the second drug binding is accelerated, suggesting that the N^CoV2^ molecules with a more flexible linker can bind the second drug molecule more quickly than those with a less flexible linker, which is a phenomenon that would affect the drug efficacy. Moreover, the promotion effects are enhanced at lower drug concentrations. On the other hand, at the lowest drug concentration (1.7 nM), ΔT was found to increase as the interdomain flexibility increases, indicating that the interdomain flexibility may serve as both an accelerator and a decelerator of drug binding. Since this simulation study is the first to investigate the potential effects of interdomain flexibility on drug binding/encountering rate, the findings would be useful to design further experimental and theoretical approaches to understand the interaction mechanism between N^CoV2^ and drugs/ligands.

Above studies emphasize the importance of experimental characterization of the molecular flexibility as well as the structures of N^CoV2^ in terms of drug binding, interaction with other viral proteins and RNA-binding. Since the molecular flexibility such as the intramolecular and interdomain flexibility mentioned above arises from thermal fluctuations of atoms in amino acid side chains and backbones [55], it is crucial to focus on the dynamical aspects at the atomic level. The detailed analysis of the structural fluctuation of the flexible proteins such as the full-length N^CoV2^ in different chemical environments is not straightforward, but experimental techniques such as NMR [56] and incoherent neutron scattering (iNS) [57] would provide valuable information in this regard. As for the structure in solution, small-angle X-ray scattering [58] is a powerful method to determine the overall structure as already employed for N^CoV2^ [20]. Further structural information could be obtained by higher-angle X-ray scattering [59].

## 3. Liquid-Liquid Phase Separation (LLPS) of N^CoV2^

The flexible linker of N^CoV2^ plays a crucial role not only in light of drug encounter/binding rate as described in the previous section, but also in forming a higher-dimensional structure that is considered important for the viral replication cycle as described below. In the past, it was shown that in the negative-sense RNA viruses such as rabies virus, cytoplasmic inclusion bodies called Negri body (NB) are formed in the infected cells, which contain the nucleocapsid protein, the RNA-dependent RNA polymerase (L), the phosphoprotein (P) and other proteins [60]. Later, it was found that the NBs show liquid-like nature and are formed by phase separation [61]. In other negative-sense RNA viruses, the similar droplets formation through the so-called liquid-liquid phase separation (LLPS) has been observed [62,63]. These structures are considered to play a pivotal role in transcription, replication and assembly of virions by concentrating proteins and RNA locally to increase the effective concentration of each component and thus accelerate the biochemical reactions. Indeed, it was shown that in measles virus, the rate of nucleocapsid assembly is accelerated when the nucleocapsid proteins are concentrated by LLPS [63]. Furthermore, it is known that P protein is essential for the formation of these droplets as well as the nucleocapsid protein [61,62,63]. On the other hand, the positive-sense RNA viruses such as SARS-CoV-2 lack the gene encoding P protein, implying that the viral proteins and RNA in this type of viruses might have developed a different strategy for their proliferation in the infected cells.

**Figure 5:**
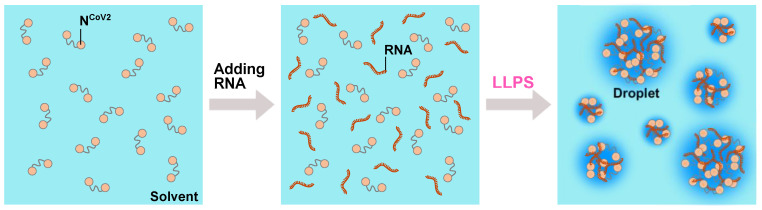
A schematic illustration of the droplet formation by N^CoV2^ and RNA. When RNA molecules are added to the N^CoV2^ solution (**left**), the mixture of N^CoV2^ and RNA (**middle**) spontaneously phase-separates into droplets (**right**), which are dynamic and fuse into larger droplets upon making contact. This phenomenon is called liquid-liquid phase separation (LLPS).

A bioinformatic study using phase separation prediction methods, however, pointed out that N^CoV2^ also possesses the ability to form condensates with RNA and key host cell proteins [64]. In line with this prediction, the latest experimental studies have revealed that N^CoV2^ form droplets with RNA, other viral proteins such as the RNA-dependent RNA polymerase (RdRp) and M protein, a stress granule protein G3BP1 and human ribonucleoproteins through LLPS at ambient temperature or even at human body temperature [43,65,66,67,68,69,70]. Furthermore, it has been shown that the flexible central linker of N^CoV2^, in particular, LQ-R (210–246), is essential for the formation of the droplets [67,68]. In addition, other flexible regions, the N-tail (1–48) and the C-tail (365–419) regions were found to enhance the droplet formation [67]. In the case of LLPS by N^CoV2^ and RNA, whose schematic illustration is shown in Figure 5, the shape of the droplets depends on the ratio of N^CoV2^ and RNA concentrations [65,66]. For example, at the fixed N^CoV2^ concentration (20 μM), liquid-like droplets are formed at the RNA (20 nt) concentrations of 2–10 μM in PBS (pH 7.4) supplemented with 2.5% PEG8000 [65]. The strongest LLPS appears to occur when charge neutralization, where the net charge of the N^CoV2^ molecules is equal to that of the RNA molecules in a solution sample under the assumption of a charge of −1 per phosphate group, has been reached, but the phase separation is suppressed as the RNA concentration further increases [66,67]. The size of the droplets was found to be affected by the length of RNA employed: as the length of RNA increases from 200 nt to 60 nt, the diameter of the droplets increases from less than 20 μm to more than 40 μm [65]. The phase separation behavior depends on pH and salt concentration as well [67]: compared with pH 7.4, more acidic pH induced stronger LLPS (at least down to pH 4.5, which was studied in [67]). At pH 7.4, droplet formation was reduced above 300 mM NaCl. These results indicate that the droplets are formed through electrostatic interactions between the positively charged N^CoV2^ and the negatively-charged RNA. Furthermore, the different RNA sequences give the similar phase diagram of the droplet formation, suggesting that the droplet formation is independent of the RNA sequence. However, as noted in [65], it is unclear whether a specific RNA sequence elicits stronger LLPS or not. Finding such an RNA sequence would be intriguing. Comparison of the structural and dynamical properties between N^CoV2^ droplets containing RNA which induces stronger LLPS and those containing RNA which induces weaker LLPS would provide further molecular insights into the physical properties of N^CoV2^ droplets together with the possible differences in the physicochemical properties such as the transcription rate between the two types of droplets.

In addition to RNA, N^CoV2^ has been shown to form condensates with the C-terminal domain of M protein (amino acids 104–222; denoted as M^104–222^) even in the absence of RNA [68]. When M^104–222^ is added to the preformed N^CoV2^/RNA droplets, it forms an annular shell on the surface of the droplets that is stable for hours at 293 K. Furthermore, when N^CoV2^, M^104–222^, and RNA (265 nt) are mixed simultaneously at a molar ratio of N^CoV2^:M^104–222^:RNA = 100:50:1, they are separated into two subdomains of N^CoV2^/RNA and N^CoV2^/M^104–222^. As a result, in the droplets formed by the three components, the core made of N^CoV2^/RNA is surrounded by a shell of N^CoV2^/M^104–222^, or vice versa. The fluorescence recovery after photobleaching (FRAP) analysis has shown that all the three components show very slow dynamics within the droplets, indicating that N^CoV2^ interacts with M^104–222^ in the droplets [68]. It is, however, unknown how they interact with each other in terms of molecular structure. In infected cells (human osteosarcoma U2OS cell line), N^CoV2^ phase-separates into condensates with RNA and the stress granule core protein G3BP1, but not with other stress granule proteins, suggesting that N^CoV2^ suppresses the G3BP1-dependent host immune system [68]. The N^CoV2^ droplets thus appear to serve not only as a scaffold to promote the viral replication but also as a system to evade the innate immune response.

The SR-R (176–206) in the central linker contains multiple serine residues, which are phosphorylatable. The corresponding region in another coronavirus IBV is known to be highly phosphorylated in infected cells [71]. Whereas the phosphorylation of this region is known to weaken the binding between N^CoV2^ (or N protein of IBV) and non-viral RNA [66,71], the phosphorylated IBV N protein binds to viral RNA with the similar affinity as the non-phosphorylated counterpart does [71]. On the other hand, dephosphorylation of N protein of the murine coronavirus, mouse hepatitis virus (MHV) has been suggested to be involved in early host-virus interactions [72]. These results suggest that phosphorylation may serve to facilitate the specific binding of N^CoV2^ to viral RNA and that tuning of the phosphorylation states is an integral part of the viral infection and replication cycle. In the case of N^CoV2^, the serine/arginine protein kinase 1 (SRPK1) was shown to generate two species, where only S188 is phosphorylated or four residues are heterogeneously phosphorylated [66]. Even a single phosphorylation at S188 significantly reduces the interaction between N^CoV2^ and RNA (polyU), suggesting that this residue is critical for that interaction. Furthermore, the SRPK1-phosphorylated N^CoV2^ has been shown to modulate the LLPS behavior of N^CoV2^ [66]: at a fixed N^CoV2^ concentration, droplets of the phosphorylated N^CoV2^ are formed at polyU concentrations around half of those required for the unphosphorylated counterpart. Inside the droplets formed by N^CoV2^ and polyU, SPRK1-phosphorylated N^CoV2^ undergoes more rapid diffusive motions than unphosphorylated N^CoV2^, corroborating the fact that the interaction between N^CoV2^ and non-viral RNA is reduced by phosphorylation in the SR-R region. In the case of the droplets formed by N^CoV2^, polyU and RdRp, the SRPK1 phosphorylation reduces the amount of RdRp incorporated into the droplets, suggesting that N^CoV2^ interacts with RdRp as well as RNA in the droplets [66].

In addition to proteins, small peptides are also known to show phase separation with RNA through electrostatic interactions [73,74,75]. It has been shown that for a system of arginine-rich peptides ({RRASL}^3^ or {SR}_4_ENLYFQG{SR}_4_) and polyU, the molecular weight of which is 600–1000 kDa corresponding to 1800 nt–3000 nt, droplets (also known as coacervates) are formed and dissolved as a function of peptide-RNA ratio [73]. In this system, when the weight percent concentration of peptides and RNA is almost equal in the low salt concentration (0–50 mM), phase separation occurred at the highest level. As the RNA concentration increased beyond this level, droplets were dissolved. Furthermore, it has been found out that the phase separation behavior of this system is controlled by divalent cation concentration such as Mg^2+^ [74]. At the Mg^2+^ concentration more than 200 mM at the peptide-RNA ratio ([peptides]/[polyU]) of 0.6 (wt/wt), heterotypic droplets (peptides-RNA droplets) were transformed into homotypic droplets (RNA-RNA droplets). Another study has shown that formation and dissolution of the peptide-polyU droplets are regulated by the phosphorylation state of the peptides [75]. It was demonstrated that the phosphorylation of serine residues in the peptides (RRASLRRASL) prevents droplet formation. Thus, fundamental properties of droplets formed by small peptides and RNA are similar to those observed for N^CoV2^ droplets, indicating that interactions occurring at the amino acid level are prerequisites for droplet formation.

Fluorescence measurements using Alexa fluorophores have shown that the N^CoV2^ droplets are dynamic objects: when N^CoV2^ or RNA molecules are added to the preformed N^CoV2^/RNA droplets, both types of molecules are incorporated into the droplets and simultaneously the N^CoV2^ and RNA molecules that constituted the preformed droplets diffuse out of the droplets, suggesting that both N^CoV2^ and RNA are exchangeable between the dense and dilute phases [65]. This also indicates that diffusive motions of both N^CoV2^ and RNA are crucial for dynamically maintaining the droplets. Moreover, when the complex of RdRp and RNA is added to the N^CoV2^/RNA droplets, the entire RdRp-RNA complex is also incorporated into the droplets [66], suggesting that the preformed droplets have the ability to further take in other viral proteins. FRAP measurements have shown that in the droplets formed by N^CoV2^/RNA and G3BP1, there are three populations of the N^CoV2^ molecules that diffuse at different mobility [66]. These results raise the possibility that the diffusive motions of the N^CoV2^ molecules in the droplets might play an important role in the transcription and replication cycle. Since the diffusion of the whole protein molecules inside the droplets is largely affected by the strength of interaction between N^CoV2^ and other proteins/RNA, it would be of great interest to understand the relevant physical properties of N^CoV2^ such as side-chain fluctuations, which directly determine the rate and the manner of interaction with their binding partners.

## 4. Perspective–Toward Structural and Dynamical Characterization of N^CoV2^ Droplets

The recent findings that N^CoV2^, RNA, other viral proteins and a stress granule protein are concentrated into a different phase from its surroundings suggest that the N^CoV2^ droplet formed through LLPS is the main arena where molecular events critical for the virus replication take place. It is thus indispensable to reveal the internal organization of N^CoV2^ droplets, especially the structure of N^CoV2^ in droplets formed with various binding partners to develop a new intervention technique targeting N^CoV2^ as well as to understand the molecular mechanism of biological functions of N^CoV2^ in detail. Furthermore, the molecules within the droplets are not frozen to the spot but fluctuate dynamically around their average conformation under the influence of thermal energy. The nature of these structural fluctuations is closely related to the interaction, the stability and the function of the proteins. Thus, unraveling the structure and structural fluctuation of N^CoV2^ in the droplets formed in various conditions is essential and the research in this direction would occupy an important position in this research field.

**Figure 6:**
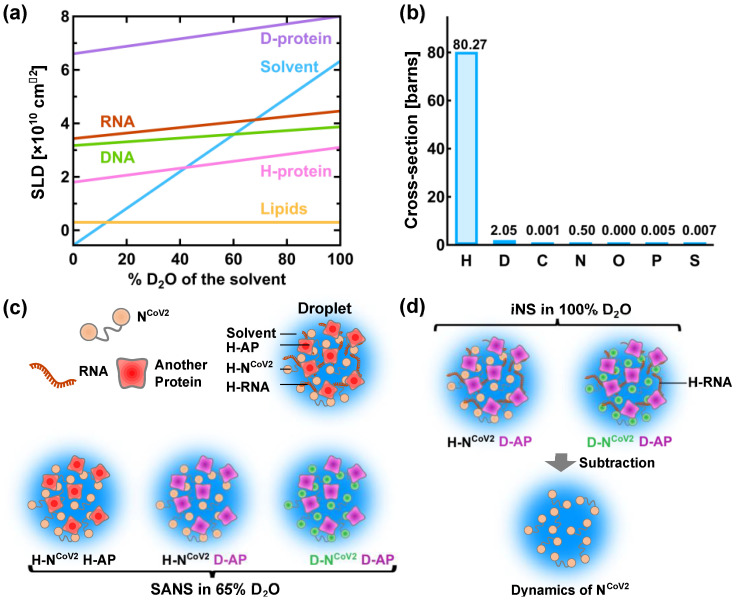
A promising method for physical characterization of the N^CoV2^ droplets using neutron scattering. (**a**) Variation of the neutron scattering length density (SLD) of biomacromolecules as a function of the heavy water concentration in the solvent. H-protein and D-protein denote the hydrogenated and perdeuterated proteins, respectively. The values of the SLD of H-protein, D-protein, lipids and solvent are taken from [81], and those of DNA and RNA are taken from [83]; (**b**) values of incoherent neutron scattering cross-section of atoms found in biomolecules and an isotope of hydrogen atom, deuterium. Note that 1 barn = 10^−24^ cm^2^. The values are taken from [92]; (**c**) schematic illustration of the structural analysis of the N^CoV2^ droplets using contrast-matching small-angle neutron scattering (SANS). The components that are “invisible” to neutron are shown in the same color as solvent. The prefixes H- and D- denote “hydrogenated” and “perdeuterated”, respectively, which are shown in different colors. AP denotes another protein; (**d**) schematic illustration of the molecular dynamics analysis of N^CoV2^ droplets using incoherent neutron scattering (iNS) combined with deuteration technique. For detailed explanations of (**c**,**d**), refer to the main text.

A powerful method to characterize the molecular structure of N^CoV2^ in droplets would be small-angle neutron scattering (SANS). This technique has been used to characterize the structure of viral proteins in isolated state [76,77] and in complex with other viral components [78,79,80]. However, to the author’s knowledge, the structural and dynamical analysis of droplets formed by virus-related proteins has not been carried out so far. Even though there are excellent reviews and books on neutron scattering [81,82,83,84], some basic information on neutron scattering is given below in order to describe a possible method to reveal the structure and dynamics of N^CoV2^ droplets. In neutron scattering, the strength of scattering (coherent scattering length) depends on the type of atoms bombarded by neutron as shown in Table 1. The major feature of coherent neutron scattering is that the scattering length of hydrogen and its isotope deuterium is largely different and thus deuteration technique plays a significant role in the structure analysis of a complex system that consists of different kinds of components [85,86,87]. In the case of the solution samples such as protein solutions, the scattering signal in SANS measurements derives from the difference (contrast) in the scattering length density (SLD: the total scattering length of a molecule divided by its volume) between solutes and solvents. Figure 6a shows the variations of the SLD of major biomolecules (proteins, lipids, DNA and RNA) as a function of the fraction of D_2_O in the solvent. As the D_2_O content in the solvent increases, the labile hydrogen atoms in the solutes are exchanged for deuterium atoms, which modifies the SLD of the solutes. The SLD of perdeuterated proteins (denoted as “D-protein” in Figure 6a) is much larger than that of the hydrogenated counterparts because all the hydrogen atoms including those in methyl and ethyl groups are replaced with deuterium atoms. As seen in Figure 6a, the SLD of the solvent containing 40% D_2_O is equal to that of the hydrogenated protein (H-protein), meaning that the scattering contrast of the H-protein is zero. In this condition, the contribution of the H-protein to the scattering signal is effectively zero and hence it is “invisible” to neutron. Even though techniques to perdeuterate or partially deuterate RNA molecules by chemical synthesis, in vitro transcription and in vivo production have been established for NMR studies [88,89], production of the sufficient amount of perdeuterated RNA for neutron experiments is more expensive and/or more difficult than production of perdeuterated protein and thus there have been only a very few SANS studies using perdeuterated RNA [82,90]. Therefore, in the following, only RNA in the hydrogenated state (H-RNA) is considered.

Figure 6c illustrates a promising method to characterize the N^CoV2^ structure in droplets. In the case where droplets are formed by N^CoV2^, RNA and another protein (Figure 6c), three kinds of samples would be required to obtain the scattering signal from each protein: (1) droplets formed by H-N^CoV2^, H-RNA and the hydrogenated another protein (H-AP), (2) those formed by H-N^CoV2^, H-RNA and the perdeuterated another protein (D-AP) and (3) those formed by the perdeuterated N^CoV2^ (D-N^CoV2^), H-RNA and D-AP. The SANS measurements in these samples in the ~65% D_2_O solvent, the SLD of which matches that of H-RNA, provide three kinds of scattering data where each protein contribution is different. From these data, the scattering data arising from each protein can be extracted, leading to the elucidation of its conformation together with how each protein is arranged inside the droplets.

The structural fluctuation of the molecules in the droplets are obtained by incoherent neutron scattering (iNS). This technique measures the intensity of neutron scattered by the samples as a function of the momentum and energy change of the neutron, from which the amplitude, the frequency and their distribution of atomic motions at pico- to nanosecond timescale at ångström length scale are estimated [57]. The values of incoherent scattering cross-section of atoms found in biomolecules are shown in Figure 6b. It is found that the scattering cross-section of hydrogen atoms is much larger than any other atom and deuterium, meaning that iNS provides information of the motions of hydrogen atoms. Furthermore, the measurements in 100% D_2_O buffer can minimize the solvent contribution to the iNS spectra. Since the hydrogen atoms are quasi-uniformly distributed throughout the molecules in the biological systems, the dynamical information averaged over the whole molecule is obtained. In the case of proteins, the observed motions reflect the fluctuations of amino acid side chains and backbones because hydrogen atoms are bound to these chemical groups. The schematic illustration of the dynamical analysis of the N^CoV2^ droplets is depicted in Figure 6d. For droplets generated from N^CoV2^, RNA and another protein, two types of samples are required: (1) droplets formed by H-N^CoV2^, H-RNA and D-AP, (2) those formed by D-N^CoV2^, H-RNA and D-AP. By subtracting the iNS spectra of the latter from those of the former measured in 100% D_2_O, the remaining spectra arise from the structural fluctuation of the N^CoV2^ molecules, thereby providing dynamical information on N^CoV2^ inside the droplets. Instead, the dynamics of another protein in the droplets can be extracted in a similar manner.

**Figure 7:**
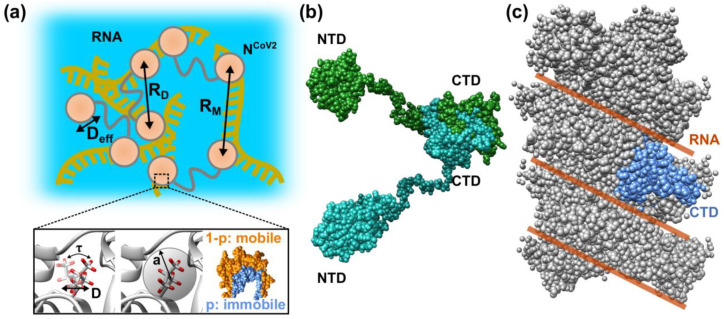
N^CoV2^ in different structural states. (**a**) Schematic illustration of structural and dynamical information on N^CoV2^ droplets that would be obtained by the methods described in Figure 6. R_D_ and R_M_ show the distance between the NTD and CTD, and the distance between domains of different N^CoV2^ molecules, respectively. D_eff_ denotes the efficient diffusion coefficient of the NTDs/CTDs and/or of the N^CoV2^ molecules. In the lower panel, τ, D, and a denote the residence time, the diffusion coefficient, and the amplitude of amino acid side chains and/or backbones, respectively. Hydrogen atoms are shown in red. p and (1-p) show the immobile and mobile fractions of atoms in proteins; (**b**) N^CoV2^ in an isolated state (dimer). Each monomer is shown in sea green and forest green, respectively. The central linker was modelled using the Ranch program [93]; (**c**) a model of nucleocapsid complex for SARS-CoV [8]. Only CTDs are displayed because NTD positions are unknown. Possible locations of RNA molecules are depicted by brown line. One CTD monomer is shown in marine blue The models in sphere or ribbon representation are depicted using UCSF Chimera [16].

That way, the structure and structural fluctuation of the proteins contained in the droplets, which play a crucial role in virus replication, are characterized. For example, following parameters characterizing the internal organization of the droplets are found: as shown in Figure 7a, the interdomain distance (R_D_) of the identical N^CoV2^ molecule and the distance between domains of different N^CoV2^ molecules (R_M_) can be determined from structural analysis. This means that not only the conformation of N^CoV2^ in droplets but also the arrangements of the N^CoV2^ molecules in droplets are revealed. From the iNS measurements, efficient diffusion coefficients (D_eff_), which include translational and rotational diffusion, of the whole N^CoV2^ molecule and/or domains will be obtained. Furthermore, as shown in the lower panel of Figure 7a, the residence time (τ), during which atoms stay in one site before moving to another site, and the diffusion coefficient (D) of this motion can be estimated. Regarding the geometry of motions, the immobile fraction of atoms (p), motions of which are too slow to be observed by the neutron spectrometer employed and the mobile fraction of atoms (1-p) are obtained. For the latter, the amplitude (a) of atomic motions is estimated. By comparing the structure of N^CoV2^ in droplets with that in an isolated state (Figure 7b) and in a nucleocapsid complex (Figure 7c), structural features that are unique to droplets and thus considered important for the virus replication/infection cycle would be revealed. The dynamics information will elucidate the mode of molecular and atomic motions unique to N^CoV2^ in droplets. It is likely that the unique structure and arrangements of proteins inside the droplets form a field where RdRp molecules bind to RNA more easily than outside the droplets, promoting the viral genome transcription. The unique mode of protein motions inside the droplets would be the determinants of interactions between other molecules inside the droplets, affecting the efficiency of genome transcription. All the above structural and dynamical information will thus be useful not only for understanding the molecular mechanism of the virus replication/infection cycle but also developing medical intervention techniques based on the structure and dynamics of proteins. Moreover, since the time- and space-scales of iNS are comparable to those of MD simulation, dynamics parameters obtained from iNS would serve as a fundamental information in in silico drug development targeting the droplets in the future.

## 5. Conclusions

In conclusion, the molecular flexibility as well as conformations of N^CoV2^ plays a critical role in drug-binding, RNA-binding and formation of the droplets. Knowledge of the structure and dynamics of the N^CoV2^ molecules in isolated states in solution and in a variety of droplets would advance our understanding of the architecture and the physical properties of the droplets, thereby shedding light on the molecular mechanism of the viral infection and replication cycle. Furthermore, all this information would eventually be beneficial to the development of new drug molecules targeting N^CoV2^, of improved diagnostic techniques, of a new type of treatment for the COVID-19 pneumonia, and of a new combat strategy for a future pandemic.

## Figures and Tables

**Table 1:** Summary of the coherent neutron scattering length (b_coh_) of atoms in biomolecules [91].

Atom	b_coh_ [10^−12^ cm]
H	−0.37409
D	0.6674
C	0.66484
N	0.936
O	0.5805
P	0.513
S	0.2847

## Data Availability

Not applicable.
